# Children’s autistic traits and peer relationships: do non-verbal IQ and externalizing problems play a role?

**DOI:** 10.1186/s13034-021-00421-2

**Published:** 2021-11-22

**Authors:** Novika Purnama Sari, Maartje P. C. M. Luijk, Peter Prinzie, Marinus H. van IJzendoorn, Pauline W. Jansen

**Affiliations:** 1 Department Psychology, Education & Child Studies, Erasmus University Rotterdam, Rotterdam, The Netherlands; 2 Department of Child & Adolescent Psychiatry/Psychology, Erasmus University Medical Centre, Rotterdam, The Netherlands; 3 Generation R Study Group, Erasmus University Medical Center Rotterdam, Rotterdam, The Netherlands; 4 Research Department of Clinical, Educational and Health Psychology, UCL, University of London, London, UK

**Keywords:** Peer acceptance, Peer rejection, Autistic traits, Nonverbal IQ, Externalizing problems

## Abstract

**Background:**

Children with autism have difficulties in understanding relationships, yet little is known about the levels of autistic traits with regard to peer relationships. This study examined the association between autistic traits and peer relationships. Additionally, we examined whether the expected negative association is more pronounced in children with a lower non-verbal IQ and in those who exhibit more externalizing problems.

**Method:**

Data were collected in a large prospective birth cohort of the Generation R Study (Rotterdam, the Netherlands) for which nearly 10,000 pregnant mothers were recruited between 2002 and 2006. Follow up data collection is still currently ongoing. Information on peer relationships was collected with PEERS application, an interactive computerized task (*M* = 7.8 years). Autistic traits were assessed among general primary school children by using the Social Responsiveness Scale (*M* = 6.1 years). Information was available for 1580 children.

**Result:**

Higher levels of autistic traits predicted lower peer acceptance and higher peer rejection. The interaction of autistic traits with externalizing problems (but not with non-verbal IQ or sex) was significant: only among children with low externalizing problems, a higher level of autistic traits predicted less peer acceptance and more peer rejection. Among children exhibiting high externalizing problems, a poor peer acceptance and high level of rejection is seen independently of the level of autistic traits.

**Conclusion:**

We conclude that autistic traits—including traits that do not classify as severe enough for a clinical diagnosis—as well as externalizing problems negatively impact young children’s peer relationships. This suggests that children with these traits may benefit from careful monitoring and interventions focused at improving peer relationships.

**Supplementary Information:**

The online version contains supplementary material available at 10.1186/s13034-021-00421-2.

## Introduction

Children with an autism spectrum disorder (ASD) experience difficulty in developing, maintaining, and understanding relationships. These range from difficulties adjusting behavior to suit various social contexts, to problems in sharing imaginative play and making friends [1]. A study by Bauminger and Kasari, [2] revealed that children with ASD actually experience a desire to have friends and interact with peers, but remain at an increased risk for social problems in regular classroom settings. Despite a number of studies on social involvement and friendships of children with ASD [3–5], there has been relatively little research examining levels of autistic traits in regards to different aspects of peer relationships, such as peer acceptance and peer rejection. Most of the previous studies were conducted in clinical samples which mostly included boys. Furthermore, little is known about the potential moderating role of cognitive functioning and externalizing problems in the association between autistic traits and peer relationships. Evaluating which characteristics affect the relation between autistic traits and peer relationships will provide crucial information for school teachers and can help defining targets for social skills training programs. The current study aims to address this gap by using data from a large population-based sample called Generation R, with unique peer reports on peer relationships, i.e. peer acceptance, peer rejection, prosocial behavior, and reciprocity.

In the field of peer relationship research, peer relationships are typically assessed with questionnaires, field notes, or interviews [6]. Most studies rely on one reporter, often the child him/herself, the teacher or one of the parents. Although peer reports may provide a more comprehensive picture of peer relationship within a classroom, obtaining peer reports in (young) children is also a challenge. Previously, a study by Fujiki et al. [7] has successfully used a picture board rating scale to avoid problems first graders might have in recognizing classmates’ names in written form. Animated, digital assessment instruments may provide another option here, although they are rarely used for research yet. Following the successful examples of studying peer relations with the help of illustrations/cartoon methodology [8], Verlinden et al. [9] developed an animated assessment instrument—the PEERS Measure. In this interactive, computerized measure, children were shown the photographs of their classmates to allow nominations. The PEERS Measure summarizes the nominations into peer acceptance and peer rejection scores, which are indicators of how well children socialize with other children within a school setting. Furthermore, a prosocial behavior score can be computed as an indicator of how often children comfort other children when they are sad, while the reciprocity score indicates whether children nominate back.

## Peer relationships among children with ASD

In the early 1990s, the majority of children with ASD were educated in special schools in the Netherlands [10]. Yet, in the last decades, educational policies have encouraged teachers and parents to place children with ASD within the mainstream schools in the Netherlands [11]. Despite a number of benefits of inclusive settings, children with high levels of autistic traits (either diagnosed ASD or subclinical levels) may be more likely to experience problems in peer relationships within the classroom [12].

Due to social impairments, children with ASD experience challenges in developing peer relationships. Previous studies [4, 5, 13–15], mostly using survey and questionnaires, have reported that children with ASD are less frequently nominated as friends by their neurotypical peers. The majority of these studies investigated peer relationships within a clinical population of children diagnosed with ASD. Yet, investigating whether autistic traits in the general population is also of significance because it allows us to understand whether children with a lower level of autistic traits also experience similar but perhaps less severe difficulties in peer relationships.

## Non-verbal IQ and externalizing problems

Although numerous studies have investigated the profile of peer relationships among children with ASD, we do not know yet whether certain characteristics affect the risk of poor social relationships with peers. Identifying such characteristics is important for intervention programs. One factor that may moderate outcomes associated with peer relationships among children with ASD is intelligence. Indeed, another study which relied on the same cohort as the current study, found that a lower intelligence quotient (IQ) has been associated with bullying involvement in early elementary school [16], and that children with a higher non-verbal IQ are less likely to be victims and bully-victims. The authors suggested that children with a higher non-verbal IQ are more skilled in either preventing peer victimization or in effective resolution of peer conflicts. In this way, a lower non-verbal IQ may reduce children’s capabilities needed for social functioning, making them more vulnerable to peer relationship problems. Additionally, another study on social relations in mid- to later adulthood among individuals diagnosed with autism showed that a higher nonverbal IQ predicts better social relations [17]. On the other hands, Hauck et al. [18] found that the number of social initiations by children with ASD to classmates was predicted by their communication ability and receptive vocabulary, but was not significantly predicted by their level of autistic traits. Verbal IQ was not measured and assessed as a predictor by Hauck et al. [18], yet verbal IQ may, along with non-verbal IQ, overlap with the constructs of communication ability and receptive vocabulary. Indeed, non-verbal and verbal IQ are not independent [19], and non-verbal IQ might be considered a better proxy than verbal or overall IQ in large samples [20].

Another factor that may moderate the association between autistic traits and peer relationships is externalizing problems. Numerous studies using parent and teacher reports showed that peer rejection is robustly associated with externalizing problem [21, 22]. A study conducted by Sturaro et al. [23] used a peer nomination method in 30 elementary schools in the Netherlands, and demonstrated that children’s externalizing problems predicted peer rejection consistently across time.

## Current study

In sum, empirical evidence suggests that children with ASD, children with a lower non-verbal IQ and children exhibiting externalizing problems experience difficulties in their peer relationships. However, many of the aforementioned empirical studies were focused on clinical samples, and—to our knowledge—no study investigated the association between autistic traits and peer relationships using a population-based design. The current study was conducted in a large population-based cohort, including boys and girls with and without (sub)clinical autistic traits. A unique feature of this cohort is the extensive data collection on peer relationships using the PEERS Measure [9], resulting in information on peer relationships based on the reports of classroom peers. The aim of this study was to test the hypothesis that children with more autistic traits will be more likely to experience less peer acceptance, more peer rejection, less prosocial behavior, and less reciprocity, as compared to children with only few or no autistic traits. Additionally, we expected that the associations between autistic traits and indicators of a poorer peer relationship quality are more pronounced in children with a lower non-verbal IQ or who have more externalizing problems. As ASD is more common in boys than girls, with a ratio of approximately four to one [24, 25], we explored whether sex moderated the association between autistic traits and peer relationships.

## Method

### Design and study participants

The study was embedded in the Generation R Study, a large population-based cohort from fetal life onward in Rotterdam, the Netherlands [26]. In short, the Generation R Study was designed to classify early environmental and genetic causes of normal and atypical growth, development and health until adulthood [27]. It enrolled 9778 mothers who had been recruited through midwives and obstetricians residing in Rotterdam with a delivery date between April 2002 and January 2006. All participants provided written informed consent living. The Generation R Study was approved by the institutional review board of the Erasmus Medical Centre. Assessment waves were conducted annually in the preschool period (0–4 years), followed by assessment waves around ages 6, 10 and 13 years. Data were collected through home-visits, repeated questionnaires and routine child health center visits. For the current study, we used the Social Responsiveness Scale [28], a parent-reported questionnaire data on autistic traits, which was collected when the children were 6 years of age (*M* = 6.1 years, *SD* = 0.4 years). Non-verbal IQ was also assessed at 6 years of age using two subtests of a Dutch IQ test: Snijders-Oomen Niet-verbale intelligentie Test–Revisie [36]. For externalizing problems, we used the Teacher Report Form (TRF 6–18; [29]) when the children were 6 years old (*M* = 6.8 years, *SD* = 1.3 years). The peer nomination procedure was also conducted when children were 7 years of age (*M* = 7.8 years, *SD* = 0.8 years) using the PEERS Measure (details below).

**Fig. 1:**
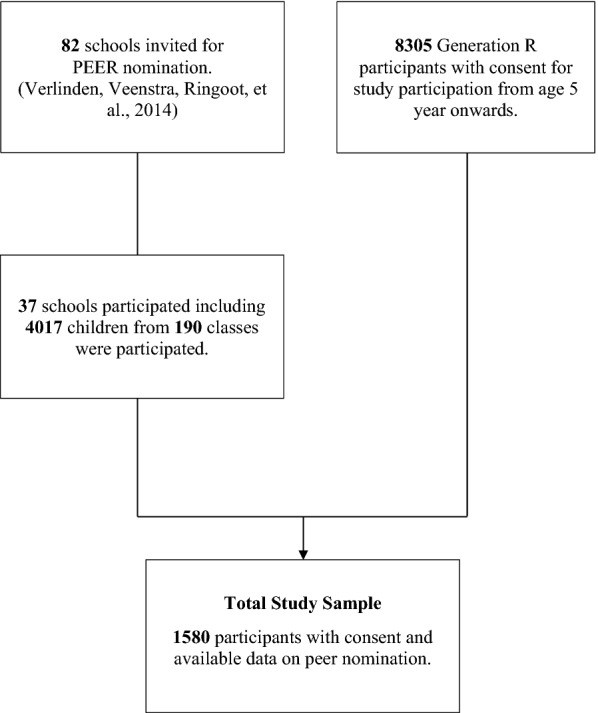
Flow chart of participants included for analysis

Eighty-two schools in Rotterdam were invited to participate in the PEERS Measure [9]. Thirty-seven schools participated over two school years (school response rate 45%), five of them more than once. For 4017 children (age 6 – 10 years) from 190 classes, parents consented for participation. Using the PEERS Measure in part of the Generation R Study cohort facilitated us to combine peer reports at school with data of Generation R participants, which were collected before the PEERS Measure was assessed. In total, 1580 of the 4017 children in the PEERS sample were also participants in the Generation R Study for whom we have information on autistic traits, externalizing problems and non-verbal IQ. Figure 1 shows the participant flowchart and Table 1 presents the baseline characteristics of the study sample.

**Table 1:** Participant characteristics (N = 1580)

Study characteristics	Total study		By sex	
	*N*	Mean (SD)	Boys (*N* = 769)	Girls (*N* = 811)
Maternal				
Education (%)				
High	787	49.8	49.5	50.1
Mid	351	22.2	22.8	21.7
Low	119	7.5	6.5	8.5
Single parenthood (% yes)	187	11.8	13.1	16.2
Autism Quotient score	817	50.1 (8.6)	50.4 (8.8)	50.6 (8.8)
Child				
Age (years)	1580	7.8 (0.7)	7.7 (0.7)	7.6 (0.7)
Sex (% boys)	769	48.7	–	–
Ethnicity (%)				
Dutch	895	56.6	55.9	57.3
Non Dutch	685	43.4	44.1	42.7
Autistic traits score	1580	3.6 (3.4)	4.7 (3.6)*	3.5 (2.9)*
Peer acceptance score	1580	0.2 (0.1)	0.2 (0.1)	0.2 (0.1)
Peer rejection score	1580	0.2 (0.1)	0.2 (0.1)	0.1 (0.1)
Prosocial behavior score	1580	0.2 (0.1)	0.2 (0.1)	0.2 (0.1)
Reciprocity score	1496	0.5 (0.3)	0.5 (0.3)	0.5 (0.3)
Non-verbal IQ	925	101.9 (14.6)	101.1 (14.8)	101.7 (14.2)
Externalizing problems score	808	2.9 (5.8)	4.7 (6.4)*	2.7 (4.3)*

Data represent means (SDs) unless specified otherwise

Autism Quotient aims to investigate whether adults of average intelligence have symptoms of autism spectrum conditions

Missing data in the total study: 20.4% Maternal education, 19.7% Single Parenthood, 48.3% Autism Quotient, 22.2% Non-verbal IQ, 31.6% Autistic Traits, 26.9% Externalizing Problems

^*^p < 0.05 for comparison between boys and girls

### Measures

#### PEERS measure

The PEERS Measure is an interactive animated web-based computer program that was used to assess peer relationships in the early school years [9]. This procedure allows children to nominate peers independently rather than in an interview. This program assesses peer acceptance, peer rejection, and prosocial behavior. It is a reliable and age-appropriate assessment that enables children aged 6–10 years to accomplish the task independently by following audio and visual instructions, and by selecting photos to answer the questions [9]. The average time needed for task completion was 7.9 min (*SD* = 1.5 min). In the validation study in Generation R: ICC_peer acceptance_ = 0.81, *p* < 0.001, ICC_peer rejection_ = 0.71, *p* < 0.001. Bland–Altman plots demonstrated good test–retest reliability [30].

#### Peer acceptance, peer rejection, prosocial behavior

The program required children to imagine they were going on a school trip. When they were prompted by the audio, they clicked on the photos of classmates that they wanted to invite on the school trip (peer acceptance) and those whom they would rather not invite on the trip (peer rejection). The following is a transcript of the instruction to the children to measure peer acceptance and peer rejection: *“Look, there is a school bus! You are going on a nice school trip to a zoo! And here are your classmates (photos of all the participating children are displayed). You can choose whom would you really like to come with you! Who would you like the most to go with you on the trip? But unfortunately, it’s not possible to take everybody along. Whom would you rather not take with you on the trip?”* Each student could nominate 0–6 classmates whom they wanted to take along, which is used to calculate a peer acceptance score, and 0–6 classmates whom they did not want to take along (peer rejection score). Then the program asked the students to nominate 0–10 classmates who are often kind to them; for instance, those who often share things with them or comfort them when they are sad. These nominations are used to calculate a prosocial behavior score.

For each peer nomination question (peer acceptance, peer rejection, prosocial behavior), individual proportions were calculated. The individual proportions represent the number of nominations given and obtained by all the other classmates, weighted by the number of classmates performing the evaluation [9].

#### Reciprocity

For each child in the classroom, a separate reciprocity score was constructed [31]. This score represents the proportion of a student’s mutually returned nominations for peer acceptance, peer rejection, and prosocial behavior. It indicates how balanced the relationships between a child and his peers are.

#### Autistic traits

Autistic traits were measured using the Social Responsiveness Scale [28]. The SRS is a widely used quantitative screening tool for autistic traits and measures the levels of autism-related traits in the general population. The SRS is an assessment of autistic behaviors in a naturalistic setting [28]. The original questionnaire was reduced to an 18-item short-form to minimize the subject burden; the short-form consists of three subscales (i.e., social communication, social cognition, social mannerism). The items are a subsample of items of the official Dutch translation of the full version [32] and they were chosen based on DSM-IV autism domains (personal communication with SRS test developer; see Additional file 1: Table S2 [33]. Parents of the children filled out the questionnaire when the children were 6 years of age (*M* = 6.1 years, *SD* = 0.4 years). Parents were asked to rate probes on a 4-point Likert scale; 0 (*not true*); 1 (*sometimes true*); 2 (*often true*); and 3 (*almost always true)*; e.g., “My child has repetitive, odd behaviors such as hand flapping or rocking”. The SRS short-form highly correlates (*r* = 0.95) with the full 65 item version [34]. Internal consistency was measured separately for males and females (Cronbach’s α for both groups: 0.92). The SRS was missing in 31.6% of the children, which was largely due to the lower response rate of the overall questionnaire (64%), which was the second questionnaire of the 6-year assessment wave. Given the moderate correlation between the SRS and the Child Behavior Checklist (CBCL, [29]) reported by parents (e.g., at 6 years: *r* = 0.33), missing values on the SRS were imputed using the total score CBCL at different ages as a predictor in the imputation model [35], for sophisticated planned missing approaches).

#### Non-verbal IQ

A standardized measure was used to assess non-verbal IQ of the children at mean age 6.0 years (*SD* = 0.85 years). Children’s non-verbal intellectual abilities were measured using two subtests of a Dutch IQ test: Snijders-Oomen Niet-verbale intelligentie Test–Revisie (SON-R 2½-7) [36]. The assessed subsets were Mosaics which assesses spatial visualization abilities of children, and Categories which assesses abstract reasoning abilities in children. Raw scores were derived from these two subtests, which were then reverted into a standardized score, based on the Dutch norm population aged 2½-7 years. The sum of the standardized scores of the two subtests was converted into a SON-R IQ score using age-specific reference scores provided in the SON-R 2½-7 manual (*M* = 100, *SD* = 15). The average retest reliability of the SON-R 2½-7 IQ score is 0.90, range 0.86–0.92 [36]. The reliability of the subtests that were used in our study are 0.73 for Mosaics and 0.71 for Categories.

#### Externalizing problems

The Teacher Report Form (TRF 6–18; [29]) was used to obtain standardized teacher ratings of children’s behavioral and emotional problems (*M* = 6.8 years, *SD* = 1.3 years). The 120-item TRF, which is the teacher version of the widely used Child Behavior Checklist [29], consists of the internalizing and externalizing problem scales. Good reliability and validity of the TRF [37, 38] were confirmed for the Dutch translation. De Groot et al [39] confirmed the applicability of Achenbach’s U.S. factor structure to Dutch children and adolescents. Verhulst et al. [40] found an average 6-week test–retest reliability for the TRF scales of *r* = 0.83. This teacher version on externalizing behavior is significantly corelated with externalizing behavior scores reported by parents at age 5 years (*r* = 0.28).

#### ASD diagnosis

To obtain an ASD diagnosis, a multiple-gating procedure was used [34]. Children’s medical records maintained by general practitioners were searched for if the data collected for the children within the Generation R Study included (a) a high score on the SRS short-form, (b) a positive score on the Social Communication Questionnaire [41] which was only administered in children with a top 15th percentile total score or 2nd percentile Pervasive Developmental Problems score on the Child Behavior Checklist (CBCL/1.5–5) or above, or (c) a parent report indicating the child had been assessed for ASD. Only children for whom a diagnosis of ASD was confirmed by these medical records were considered ASD cases in the analyses. Within Dutch clinical practice, the specialist diagnoses of ASD are generally based on clinical consensus by a multidisciplinary team. The standard diagnostic work-up involves an extensive developmental case history obtained from parents as well as school information and repeated observations of the child [34].

### Confounders

Several sociodemographic variables (child sex, ethnicity, and age; maternal educational level; maternal autism quotient; single parenthood) were considered as possible confounders, because they were previously linked with peer relationships [9, 31, 42, 43]. Information on sex and age of the child was obtained from the medical records completed by community midwives and obstetricians at birth. The child’s ethnicity was classified by the countries of birth of the parents, according to the Dutch Standard Classification Criteria of Statistics Netherlands [44]. Maternal education, defined by the highest attained education, was divided into: low education, consisting of no education and primary school only; medium education, which included secondary school level; high education, including higher vocational training and university level. Information on marital status of the pregnant women (married/cohabiting, single motherhood) was obtained by questionnaire during early pregnancy. Maternal traits of autism were obtained using the Dutch version of the Autism Spectrum Quotient (AQ-Short) [45].

### Statistical analyses

Linear regression analyses were performed in SPSS version 24 (IBM Corporation) to test our research questions. First, we examined the association between autistic traits and peer relationships separately for peer acceptance, peer rejection, prosocial behavior, and reciprocity. We constructed two models for each outcome. The first model was unadjusted. In the second model, child sex, national origin, age, maternal educational level, autism quotient, and marital status were added as covariates.

Second, we examined whether non-verbal IQ, externalizing problems, and sex moderated the associations between autistic traits and peer relationships by adding interaction terms (i.e. autistic traits by non-verbal IQ interaction, autistic traits by externalizing problems interaction, autistic traits by sex) to the regression analyses. These two potential moderators were standardized and were added in separate analyses.

**Fig. 2:**
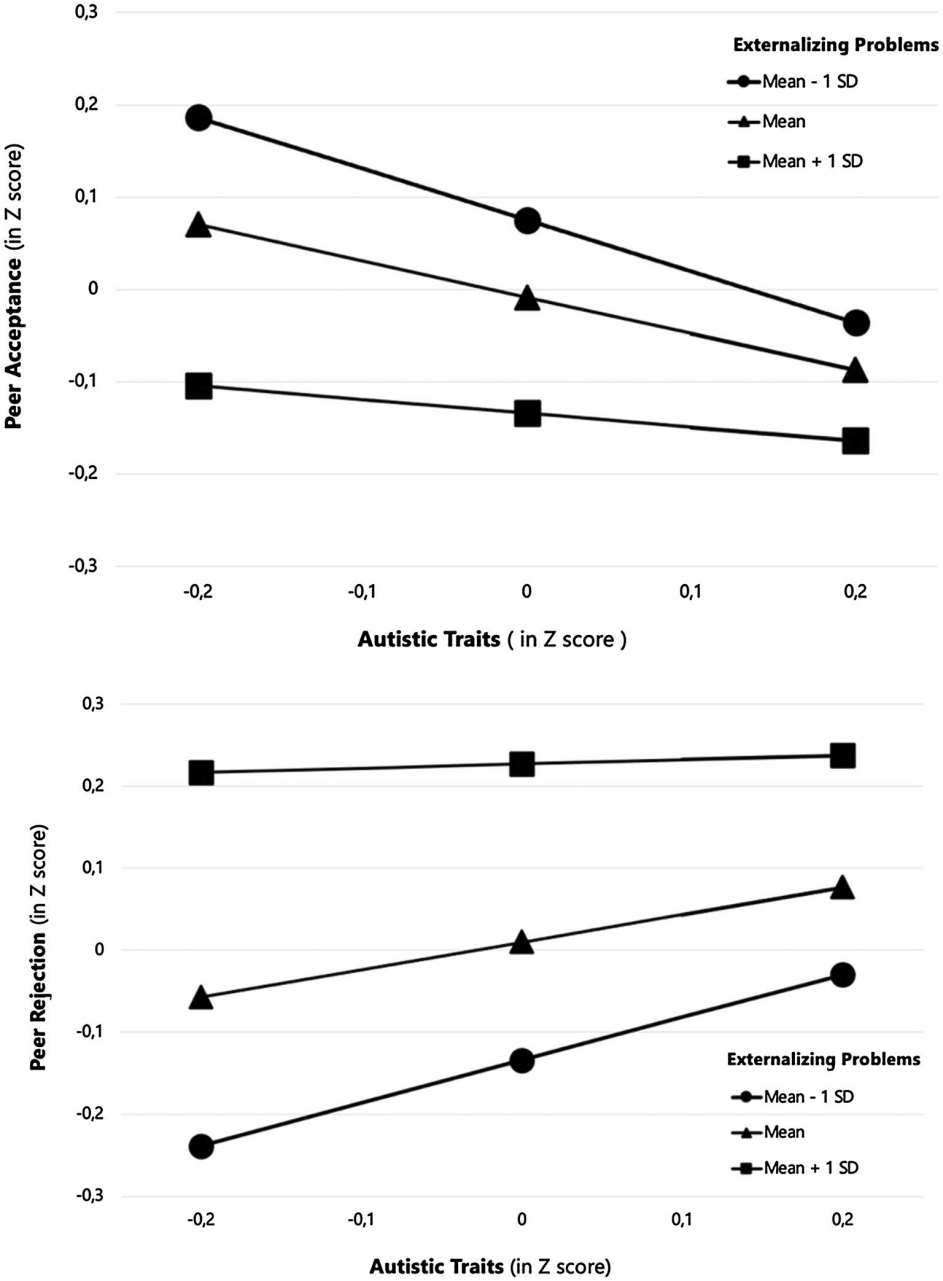
Interaction effects between autistic traits and externalizing problems on peer acceptance and rejection (N = 1580)

All scores in all analyses, including for visualization of interaction effect (see Fig. 2), were transformed into z-scores (with our own sample as reference) in order to allow comparison over different instruments and were stratified into medium (mean), low (mean – 1 SD), and high (mean + 1 SD). For missing data on autistic traits and confounders, multiple imputation was used. Twenty imputed datasets were generated using multiple imputation, and pooled estimates were calculated. The imputation method was based on linear regression used for scale variables and logistic regression for categorical variables. The variables with missing values were child autistic traits (31.6%), externalizing problems (7.6%), non-verbal IQ (3.0%), and maternal education (2.8%), marital status (4.5%) and maternal autism quotient (19.9%). These data were missing completely at random tested by Little’s MCAR test (χ^2^ (26) = 33.7, *p* = 0.07). Variables included as predictors of the imputation model were paternal education, maternal and paternal age, family income, gestational age, child sex, birth weight, and maternal IQ, total behavior problems scores reported by mothers at 18 months, 3 years (both CBCL/1½-5), and at 9 years (CBCL/6–18).

In a sensitivity analysis, we repeated the analyses on the sample after excluding those with missing data on autistic traits (*N* = 1,080 in the sensitivity analysis). Additionally, we ran a post-hoc analyses focusing on the three subscales of the SRS.

## Results

### Sample characteristics

Child and maternal characteristics are presented in Table 1. Peer relationships were assessed at the mean age of 7.8 years (*SD* = 0.8 years). Of the sample, 48.7% were boys. 56% of the children were of Dutch background. Of the mothers in our sample, 49.8% had a high educational level (higher vocational training and university level) and 11.8% were single parent. Ten children in the current sample had a confirmed clinical ASD diagnosis. The correlations between all study variables are presented in Additional file 1: Table S1.

### Association between autistic traits and peer relationships

The result of our regression analyses on the association of autistic traits and peer relationships is shown in Table 2. More autistic traits predicted lower peer acceptance (*β* = − 0.096, *p* < 0.01). This association remained statistically significant in the fully adjusted model (*β*_*adjusted*_ = − 0.091, *p* < 0.01). More autistic traits also predicted higher peer rejection (*β* = 0.158, *p* < 0.01), independently of the confounders included in the fully adjusted model (*β*_*adjusted*_ = 0.099, *p* < 0.01). Similar results were found with autistic traits being associated with less prosocial behavior (*β* = − 0.089, *p* < 0.01) and less reciprocity (*β* = − 0.110, *p* < 0.01) in the unadjusted model. However, these later associations attenuated to non-significance in the adjusted models (prosocial behavior *β* = − 0.061, *p* > 0.05, reciprocity *β* = − 0.076, *p* > 0.05).

**Table 2:** Relationships between Autistic Traits and Peer Relationships (N = 1,580)

Autistic traits (per SD)	Peer acceptance	Peer rejection	Prosocial behavior	Reciprocity
	β (95% CI)	β (95% CI)	β (95% CI)	β (95% CI)
Model 1^a^	**− 0.096 [− 0.151; − 0.042]**	**0.158 [0.103; 0.213]**	**− 0.089 [− 0.143; − 0.035]**	**− 0.110 [− 0.168; − 0.053]**
Model 2^b^	**− 0.091 [− 0.151; − 0.030]**	**0.099 [0.039; 0.160]**	**− **0.061 [**− **0.120; 0.003]	**− **0.076 [**− **0.142; 0.009]

*β* standardized beta, 95% *CI*  95% confidence interval

Proportion explained variance = 1.2% (peer acceptance), 7.8% (peer rejection)

Bold denotes statistically significant (*p* < 0.05)

^a^Unadjusted Model

^b^Adjusted model with confounders: child age, child gender, child ethnicity, maternal education, single parenthood, and maternal autism quotient

To investigate whether the associations among autistic traits and peer relationships were moderated by non-verbal IQ, externalizing behavior, and sex, we tested interaction effects. The interactions of autistic traits with non-verbal IQ and of autistic traits with sex were not significant for any of the peer relationships outcomes (peer acceptance, peer rejection, prosocial behavior, reciprocity). Only for externalizing problems, significant interaction effects with autistic traits were found for the peer acceptance (*β*_*interaction*_ = 0.059, *SE* = 0.33, *CI* 95% 0.011; 0.107, Cohen’s f^2^ = 0.2) and peer rejection scales (*β*_*interaction*_ = − 0.050, *SE* = 0.02, *CI* 95% − 0.098; − 0.002, Cohen’s f^2^ = 0.2). Figure 2 illustrate the interaction effect between autistic traits and externalizing problems on peer acceptance and peer rejection.

Stratified analyses showed that, in children with low (mean − 1 SD) or medium (mean) level of externalizing problems, the relation between autistic traits and peer acceptance was negative and significant, meaning that among these children, a higher level of autistic traits predicted less peer acceptance. Conversely, for children with a high (mean + 1 SD) level of externalizing problems, the level of peer acceptance was relatively low for all children and not related to the level of autistic traits.

A similar pattern was observed for peer rejection: in children exhibiting more externalizing problems, the level of peer rejection was relatively high and as suggested by the non-significant association, was not associated with autistic traits. For children with low (mean—1 SD) or medium (mean) level of externalizing problems, more autistic traits predicted significantly more peer rejection.

### Sensitivity analyses

A sensitivity analysis was conducted in the sample with complete data on autistic traits (*N* = 1080). The results remained essentially the same, with autistic traits predicting lower peer acceptance (*β* = − 0.09 *p* < 0.01) and higher peer rejection (*β* = 0.10, *p* < 0.01). Similar interaction patterns were found as well (Additional file 2: Fig. 1).

### Post-hoc analyses

A post hoc analyses focusing on the three subscales of the SRS showed that social communication significantly predicted peer acceptance (*β* = − 0.09 *p* < 0.01) and peer rejection (*β* = 0.12 *p* < 0.01).

## Discussion

The current study is the first population-based cohort study examining the association of the full spectrum of autistic traits with peer relationship characteristics in middle childhood. Results suggested that higher levels of autistic traits among 6-year old children predicted lower peer acceptance and higher peer rejection at age 7 years. This was specifically true for children with few externalizing problems, whereas among children exhibiting many externalizing problems, the level of peer rejection was relatively high for all children and did not depend on the level of autistic traits. A similar moderation effect was found for peer acceptance. Associations between autistic traits and peer relations were not moderated either by non-verbal IQ or sex.

Our findings on the association of autistic traits with peer acceptance and peer rejection resonate with the existing literature on clinical populations with ASD [13]. These results suggest that the association of autistic traits with peer acceptance and rejection reflects a graded association which is also visible in the general population. It may be that children with mild autistic traits have difficulties, comparable to those with clinically diagnosed ASD, communicating and interacting with peers which lead to impairments in relationships [46, 47]. In other words, it could be due to the lack of social communication and social interaction. Consequently, these difficulties may hinder children with autistic traits to perform some key behavioral elements of relationships such as looking at someone you are talking to, listening when someone speaks and responding to their words [48, 49]. We therefore ran a post hoc analyses focusing on the three subscales of the SRS and it shows that social communication is the subscale that is significantly predict peer acceptance and peer rejection. Another possible explanation may lie in deficits in what is variously referred to as “theory of mind” (ToM; [50]) or “mentalizing” [51]. These concepts refer to the ability to understand other people’s minds, to decode their intentions, emotions and thoughts. Impaired in this ability, children with autistic traits feel confused and fail to understand other children’s behavior [52, 53] and experience deficits in empathy [54, 55]. Theory of mind, consecutively, has been shown to be positively related to high quality of interactions among peers [56]. This idea, nevertheless, needs to be examined in future research, to determine whether (mild) impairments in theory of mind can serve as a mediator in our observations that children with a higher level of autistic traits were less accepted and more rejected by their peers.

There are several possible explanations for the finding that autistic traits were not associated with prosocial behavior and reciprocity. Firstly, other studies also found that the prosocial behavior was similar in children with ASD and typically developing children [57]. Potentially, prosocial behavior can be learned as suggested by studies that showed an increase in prosocial behavior through exposure to social stories [58] or simulated ball games [59]. Furthermore, prosocial behavior may be determined by other factors such as age and [60, 61], empathy and gratitude [62, 63], and even biological characteristics, e.g., related to the oxytocin receptor [64]. Particularly, oxytocin facilitates social behavior through enhancing recognition of emotion in facial expressions and increases the level of in-group trust (see Van IJzendoorn and Bakermans-Kranenburg [65], for a meta-analysis) and was also indirectly associated with prosocial behaviors via emphatic concern and perspective taking tendencies [64].

Our finding that autistic traits do not predict reciprocity might be due to the fact that reciprocity is less stable in early childhood than later in life [66]. This may translate into children choosing different peers for the different nomination parts. Another perspective is that there seems to be a difference between how children with autistic traits see themselves, and how others see them. Many typically developing children appear to view their friendships with children with autistic traits as qualitatively different from their other friendships [4], but this may not necessarily affect their nominations. This implies a positive message that peer relationships of children with autistic traits can be developed successfully by applying an effective program in regular, inclusive classrooms.

We found an interaction effect between autistic traits and externalizing problems in predicting peer acceptance and peer rejection. Children with high levels of externalizing problems were those who were least accepted and most rejected, and surprisingly, we found that externalizing problems seemed more relevant for peer problems than autistic traits. Of course, this might be caused by measurement issues in the SRS and/or CBCL but it suggests that children with externalizing problems refers to a cluster of problems that are characterized by outward behavior, which may annoy those in the immediate environment. For that reason, peers may reject children who are invariably exhibiting behavior problems in the classroom such as getting into many fights, or being aggressive or impulsive. Furthermore, Boer and Pijl [13] showed in their cross-sectional study on attention deficit hyperactivity disorder (ADHD) and ASD that children with ADHD were least accepted and were more rejected by their peers compared to children with ASD. As ADHD behaviors in part and broadly can be classified as externalizing problems [67], these findings together suggest that children with externalizing problems are more vulnerable for being rejected compared to children with ASD. High levels of externalizing problems might overshadow the potential influence of autistics traits even when a child has a high level of autistic traits. On the other hand, in children with low levels of behavior problems, the presence and levels of autistic traits predicted lower peer acceptance and more peer rejection. In the absence of prominent and disturbing behavior problems, higher levels of autistic traits do predict poorer peer relationships.

Results of the current study indicate that non-verbal IQ did not interact with autistic traits on any peer relationships outcome. Potentially, an interpretation for the fact that we did not find an interaction for IQ might be that we used a measure of non-verbal IQ rather than verbal IQ or a combination of both. Verbal IQ may be more relevant because it measures written and oral language including vocabulary, word fluency and classification of words which probably give an advantage for social relationships. But non-verbal and verbal IQ are not independent [19], and non-verbal IQ might be used as a proxy of overall IQ in large samples and among individuals with communicative problems (e.g., children with autism; child from recently immigrated families). Another explanation might be that the range of non-verbal IQ in our sample was mostly average to relatively high. Therefore, the combination of low non-verbal IQ and high autistic traits were not represented.

## Limitations

Our study also has some potential limitations that should be considered. First, the PEERS method assesses peer relationships in a classroom setting. As a result, nominations are restricted to the (participating) children from the same classroom, whereas it was not feasible to qualify peer relationships outside the classroom. It could, however, be argued that at a young age, children spend most of their time at school rather than at sport or hobby clubs, making the peer relationships measured at school a proper one [68, 69]. Secondly, our study is limited by the fact that our sample consisted of parents with a relatively high educational level. This selective response may, however, be more relevant for prevalence studies than for association studies [70] although generalizability is restricted. Third, language might be a potential moderator of the association between autistic traits and peer relationships [71], however in this study we only have a measure on receptive language skill but no productive language skill which are more presumed to influence peer relationship. Finally, the results of this study were based on a largely non-clinical population. Although a few children in the current sample had a confirmed ASD diagnosis (*N* = 10), this sample was too small to verify the study’s findings on (sub-)clinical ASD traits. The low rate of confirmed ASD in our study corresponds to existing reports on ASD prevalence in the Netherlands [72, 73]. Therefore, we cannot generalize the findings to children with clinical ASD. Despite these limitations, our findings represent one of the few studies that examined children with varying degrees of autistic traits from the general population. Additionally, we applied a multi-informant procedure including parents, teachers, and children as informants. As peer outcomes were based on peer nominations, with scores reflecting the ratings of preferences of certain class mates, these measures were not likely subject to the usual biases.

## Future directions and implications

We expected that children with autistic traits and externalizing problems would have much more difficulties in peer relationships than children with problems in only one domain or than children without any problems. Surprisingly, we found that externalizing problems seemed more relevant for peer problems than autistic traits. Together, these findings indicate that children with autistic traits may benefit from intervention programs that focus at improving peer relations in inclusive classrooms, and that particularly children with externalizing problems are an important target group for interventions like the UCLA PEERS Program (not to be confused with our PEERS assessment) [74] or the KiVa anti-bullying program [75]. The PEERS intervention program, which focuses on improving friendships and social skills, has been tested by Schohl et al. [76] among children with ASD, indicating that the program also significantly decreased children’s problem behaviors (i.e. aggressive acts, poor temper control, sadness, anxiety, fidgeting and impulsive acts). These are the problems that predicted poor relationships most convincingly in our study.

In addition, for future studies, we recommend data collection with a longer follow up preferably until the end of primary school (8–11 years old) and perhaps even into secondary school (12–18 years old). This would yield useful information to determine the replicability of any changes that occur during the school years, for example the role of age, verbal skills and the transition to another school. The predictor (autistic traits) and the moderators (non-verbal IQ and externalizing behavior) were assessed a year before the outcome (peer relationships), which helps to establish a temporal link and may inform future studies aiming to establishing causality. Finally, we recommend that in future studies internalizing problems will also be examined as moderators.

In conclusion, children with autistic traits experience less peer acceptance and higher peer rejection. As such, the results of our study in the general population suggest that even lower levels of autistic traits–traits that do not classify as sufficient for a clinical diagnosis–impact young children’s peer relationships. What is more, our findings suggest that preventive measures should to be adapted particularly for children with externalizing behavior problems. Moreover, the findings may help school teachers identifying, in an inclusive classroom setting, children with high levels of autistic traits and externalizing problems as those who need most support with peer relationships.

## Supplementary Information

**Additional file 1:** **Table S1.** Pearson correlation coefficients among variables. **Table S2.** The Social Responsiveness Scale (SRS), short form.

**Additional file 2:** **Figure S1.** Interaction Effects between Autistic Traits and Externalizing Problems on Peer Acceptance and Rejection (*N* = 1080).

## Data Availability

The datasets analyzed during the current study are not publicly available due to the terms and conditions participants agree to when they participate in Generation R, but are available from the corresponding author on reasonable request.
