# Supplementary material for: Children’s autistic traits and peer relationships: do non-verbal IQ and externalizing problems play a role?
Source: Child Adolesc Psychiatry Ment Health. 2021 Nov 22;15:67. doi: 10.1186/s13034-021-00421-2 (PMC8609782; doi:10.1186/s13034-021-00421-2)
Supplement: Supplementary file 1 — Additional file 1: Table S1. Pearson correlation coefficients among variables. Table S2. The Social Responsiveness Scale (SRS), short form. [file 13034_2021_421_MOESM1_ESM.docx]

**Supplementary Table 1**

*Pearson correlation coefficients among variables.*

|  | 1. | 2. | 3. | 4. | 5. | 6. | 7. | 8. | 9. | 10. | 11. | 12. | 13. |  |
| --- | --- | --- | --- | --- | --- | --- | --- | --- | --- | --- | --- | --- | --- | --- |
| 1. Autistic Traits score |  |  |  |  |  |  |  |  |  |  |  |  |  |  |
| 2. Peer Acceptance score | **-.099** |  |  |  |  |  |  |  |  |  |  |  |  |  |
| 3. Peer Rejection score | **.159** | **-.409** |  |  |  |  |  |  |  |  |  |  |  |  |
| 4. Prosocial Behavior score | **-.114** | **.666** | **-.342** |  |  |  |  |  |  |  |  |  |  |  |
| 5. Reciprocity score | **-.091** | **.649** | **-.308** | **.387** |  |  |  |  |  |  |  |  |  |  |
| 6. Child Age (years) | .040 | .053* | -.011 | .063* | **.122** |  |  |  |  |  |  |  |  |  |
| 7. Child Sex (0=Boy; 1=Girl) | **-.169** | .042 | **-.171** | **.281** | .026 | -.030 |  |  |  |  |  |  |  |  |
| 8. Child Ethnicity (0=Dutch; 1=Non Dutch) | **.169** | -.017 | .098* | .033 | **-.098** | -.010 | -.014 |  |  |  |  |  |  |  |
| 9. Maternal Education (0=Low; 1=Med; 2=High) | **-.195** | .037 | **-.147** | .000 | **.103** | .059* | -.022 | **-.236** |  |  |  |  |  |  |
| 10. Single Parenthood (0=No Partner; 1= Partner) | -.038 | .037 | **-.100** | .015 | .**082** | .026 | -.043 | -.**097** | **.119** |  |  |  |  |  |
| 11. Maternal Autism score | **.257** | -.034 | -.029 | -.028 | -.028 | .011 | .003 | **.239** | **-.119** | -.058 |  |  |  |  |
| 12. Child Non-verbal IQ | **-.114** | .072* | **-.161** | .044 | .110 | -.047 | .020 | **-.207** | **.162** | .**099** | -.030 |  |  |  |
| 13. Externalizing Behavior | **.218** | **.153** | **.305** | **-.164** | **-.187** | .062* | **-.221** | .**082** | **-.092** | **-.193** | -.010 | **.328** |  |  |

Bold denotes significant at p <.001, *significant at p <.05.

**Supplementary Table 2** The Social Responsiveness Scale (SRS), short form.

|  | **My child** | **Item Number in**  **the 65-item SRS** |
| --- | --- | --- |
| **1** | Is unable to pick up on any of the meaning of conversations of older children or adults. | 10 |
| **2** | Is slow or awkward in turn-taking interactions with peers. | 13 |
| **3** | Is able to understand the meaning of other people’s tone of voice and facial expressions. | 15 |
| **4** | Avoids eye contact, or has unusual eye contact. | 16 |
| **5** | Does not attempt to interact with the other children when on the playground or in a group with other young children. | 18 |
| **6** | Has strange ways of playing with toys. | 20 |
| **7** | Has more difficulty than other children with changes in his/her routine. | 24 |
| **8** | Is regarded by other children as odd or weird. | 29 |
| **9** | Has trouble keeping up with the flow of a normal interaction with other children. | 35 |
| **10** | Has difficulty “relating” to peers. | 37 |
| **11** | Has a restricted (or unusually narrow) range of interests. | 39 |
| **12** | Is imaginative, good at pretending (without losing touch with reality). | 40 |
| **13** | Has repetitive, odd behaviors such as hand flapping or rocking. | 50 |
| **14** | Responds to clear, direct questions in ways that don’t seem to make any sense. | 51 |
| **15** | Talks to people with an unusual tone of voice (for example, talks like a robot or like he/she is giving a lecture). | 53 |
| **16** | concentrates too much on parts of things rather than ”seeing the whole picture” (for example, spins the wheels of a toy car, but doesn’t play with it as a car, or plays with doll’s hair but not with the whole doll). | 58 |
| **17** | Is inflexible, has a hard time changing his/her mind. | 61 |
| **18** | Gives unusual or illogical reasons for doing things. | 62 |
