# Supplementary material for: Children’s autistic traits and peer relationships: do non-verbal IQ and externalizing problems play a role?
Source: Child Adolesc Psychiatry Ment Health. 2021 Nov 22;15:67. doi: 10.1186/s13034-021-00421-2 (PMC8609782; doi:10.1186/s13034-021-00421-2)

**Supplementary Figure 1**


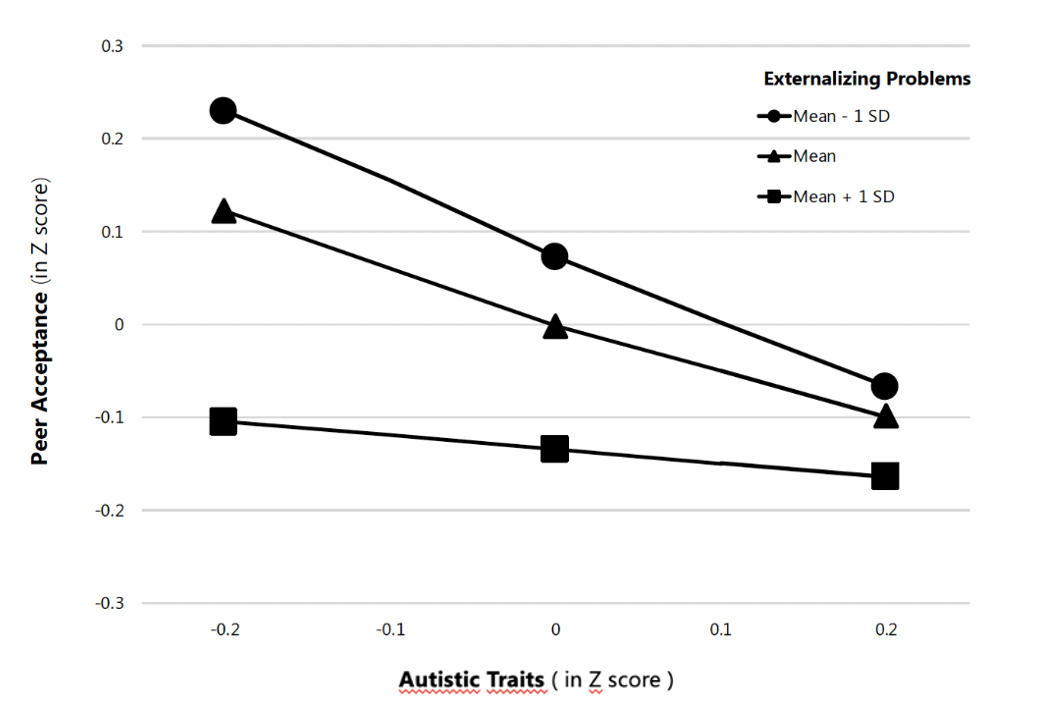
*Interaction Effects between Autistic Traits and Externalizing Problems on Peer Acceptance and Rejection (N= 1080)*


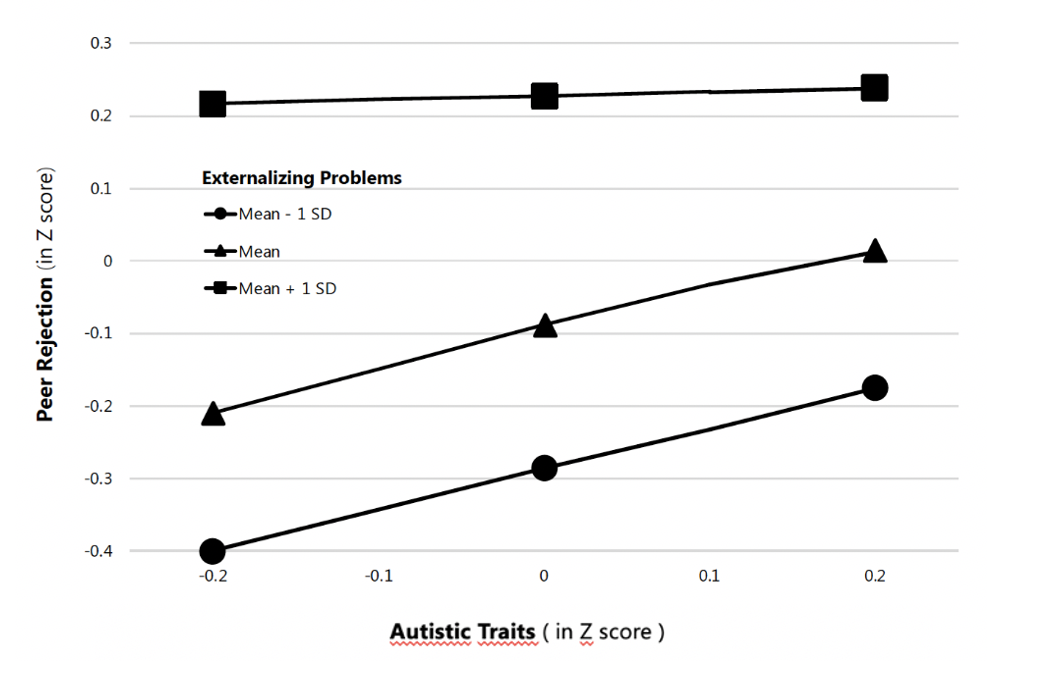

Supplement: Supplementary file 2 — Additional file 2: Figure S1. Interaction Effects between Autistic Traits and Externalizing Problems on Peer Acceptance and Rejection (N = 1080). [file 13034_2021_421_MOESM2_ESM.docx]
